# Recent developments in multifunctional neural probes for simultaneous neural recording and modulation

**DOI:** 10.1038/s41378-022-00444-5

**Published:** 2023-01-04

**Authors:** Hongbian Li, Jinfen Wang, Ying Fang

**Affiliations:** 1 CAS Key Laboratory for Biomedical Effects of Nanomaterials and Nanosafety, CAS Center for Excellence in Nanoscience, National Center for Nanoscience and Technology, Beijing, 100190 China; 2 CAS Center for Excellence in Brain Science and Intelligence Technology, Institute of Neuroscience, Chinese Academy of Sciences, Shanghai, 200031 China

**Keywords:** Biosensors, Biosensors

## Abstract

Neural probes are among the most widely applied tools for studying neural circuit functions and treating neurological disorders. Given the complexity of the nervous system, it is highly desirable to monitor and modulate neural activities simultaneously at the cellular scale. In this review, we provide an overview of recent developments in multifunctional neural probes that allow simultaneous neural activity recording and modulation through different modalities, including chemical, electrical, and optical stimulation. We will focus on the material and structural design of multifunctional neural probes and their interfaces with neural tissues. Finally, future challenges and prospects of multifunctional neural probes will be discussed.

## Introduction

A central goal in both basic and translational neuroscience is to understand the neural circuit mechanisms underlying complex behaviors^1,2^. In the brain, neural circuits process information through a temporal sequence of bioelectrical pulses from neurons^3^, which involves the inward and outward flow of ions through transmembrane protein channels^4^. Neural probes, which transduce extracellular ionic currents into electrical signals, provide a bridge between the biotic and abiotic world and have been one of the most applied tools in neural activity recording^5^. Over the past decades, neural probes with different materials and structures have been developed to record activity from large groups of neurons at single-cell resolution^6–8^. In particular, the transition from rigid to soft neural probes has greatly improved the interface between neural probes and brain tissues, enabling stable neuronal activity recordings over long periods of time^9–12^.

The central nervous system of mammals consists of various types of neurons. To unravel the causal relationships between neural circuit activity and brain function, it is necessary to simultaneously record and manipulate the activity of specific types of neurons in the brain. Over the past few years, a variety of multifunctional neural probes with integrated recording and stimulating units have been developed^13–16^. Multifunctional neural probes provide a powerful platform to simultaneously monitor the activities of neurons and their responses to well-controlled stimuli^17–19^. In this review, we summarize recent developments in multifunctional neural probes, with an emphasis on their structural and material designs. Multifunctional neural probes with different modulation modalities, including chemical, electrical, and optical stimulation, will be highlighted.

## Multifunctional probes for simultaneous neural recording and chemical delivery

Neuromodulator administration has been widely used to modulate neural activities and treat neurological disorders in medicine. Neuromodulators are generally infused by intravenous and oral administrations, which have a limited ability to reach a targeted brain region due to the presence of the blood‒brain barrier^20^. Moreover, neuromodulator drugs in the bloodstream may cause deleterious side effects and affect important physiological functions, such as memory and learning^21^. Direct brain infusion is the most effective method to deliver neuromodulators into the brain. Direct brain infusion has been accomplished by microinjection through glass/steel capillaries^22,23^. However, this technique cannot record neural activity responses to neuromodulators and thus lacks the capability to reveal the functional roles of neuromodulators.

**Fig. 1:**
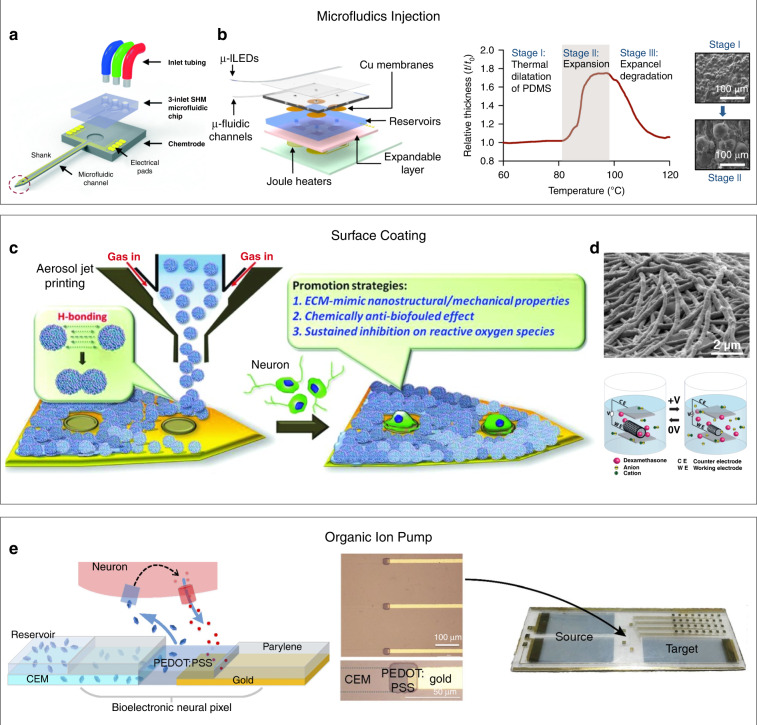
Neural activity modulation with chemical delivery. Neural activity modulation with chemical delivery. **a** Schematic of a multifunctional neural probe with integrated microfluidic chips. Reproduced with permission from ref. ^25^. Copyright 2015, The Royal Society of Chemistry. **b** A wireless fluidic probe driven by Joule heating-induced polymer expansion. Reproduced with permission from ref. ^33^. Copyright 2015, Cell press. **c** Surface coating for sustained drug release. Reproduced with permission from ref. ^36^. Copyright 2019, John Wiley and Sons. **d** Electrodes with dexamethasone/PEDOT:PSS hybrid nanotubes and drug release with electrical stimulation. Reproduced with permission from ref. ^38^. Copyright 2006, John Wiley and Sons. **e** Schematic and structure of an organic electronic ion pump-based delivery/sensing neural interface. Reproduced with permission from ref. ^41^. Copyright 2016, National Academy of Sciences

To address the above issue, drug delivery systems have been integrated with recording electrodes to achieve simultaneous neuromodulator delivery and neural activity monitoring. Initial efforts were made by integrating microfluidic channels into neural probes for localized delivery of neuromodulators into targeted brain regions^24–26^. For example, Shin et al. developed a multifunctional neural probe by integrating a 3-inlet polydimethylsiloxane (PDMS) microfluidic device with a silicon-based neural probe (Fig. 1a)^25^. To achieve a rapid mixing of drugs from different inlets, they included a staggered herringbone mixer into the microfluidic device for the delivery and mixing of multiple drugs into the brain. The multifunctional probe allowed real-time recording of neural activity responses to neuromodulators.

Although multifunctional probes based on rigid materials, such as silicon, can allow for simultaneous neural activity recording and local drug delivery, the rigidity of the probes can cause chronic inflammatory responses in the brain. For example, activated microglial and glial cells can form a compact and insulating sheath around rigid probes, resulting in signal degradation in long-term studies^27^. Compared to rigid probes, flexible probes that are integrated on polymer substrates, such as polyimide (PI)^28,29^, SU-8^30,31^, and PDMS^32^, can form more compliant probe-brain interfaces, resulting in improved long-term stability. For example, Altuna et al. developed a flexible multifunctional neural probe by combining microfluidic channels with an SU-8-based flexible neural probe^24^. The fluidic outlets were located in close proximity to the recording electrodes, which enabled simultaneous drug delivery and electrical recording of neural activity responses in the rat hippocampus at a resolution of a few hundred microns.

Fluid delivery is generally achieved by using a pump equipped with connection tubes, which may limit their applications in freely moving animals. To address this issue, Jeong et al. developed a wireless flexible fluidic probe that could be remotely controlled in awake, freely moving mice (Fig. 1b)^33^. The device consisted of two bonded PDMS layers to form a microchannel with a cross-sectional area of only 10 × 10 µm^2^. Fluid pumping was wirelessly controlled by a battery-powered infrared module. Upon activation, a thermally responsive layer beneath the reservoir was heated and expanded, resulting in drug delivery in freely moving animals. Recently, Qazi et al. developed a multifunctional probe integrated with refillable and disposable plug-n-play cartridges, which allowed prolonged drug delivery for over 4 weeks^34^.

Chemicals and drugs can also be directly loaded onto implantable neural probes for sustained release in the brain^35^. For example, Huang et al. fabricated a multifunctional neural probe by coating anti-inflammatory/anti-fouling nanogels on the surface of a neural probe (Fig. 1c)^36^. The nanogel-coated neural probe showed improved mechanical compliance with the brain tissue and thus allowed stable neural recording from rat thalamic nuclei for over 4 weeks. In addition, drugs could be selectively deposited together with conducting polymers, such as poly(pyrrole) (PPy)^37^ and poly (3,4-ethylenediocythiophene) (PEDOT)^38^, onto the recording sites of neural probes. These polymers could be electrically actuated, enabling controlled release of the loaded drugs (Fig. 1d). Nevertheless, it is difficult to control when and where the drugs are released.

Organic electronic ion pumps have been recently integrated into multifunctional neural probes for controlled drug delivery. An organic electronic ion pump consisted of two electrolyte reservoirs and a separating ion-conductive PEDOT:PSS film^39^. When a voltage was applied to the device, K^+^ ions were electrophoretically transported from the source electrolyte to the target reservoir through the PEDOT:PSS film. The local increase in K^+^ ions depolarizes the cell membrane and activates the Ca^2+^ channel to stimulate cell activity^40^. Jonsson et al. developed a 32-channel multifunctional neural probe by integrating a PEDOT:PSS-based recording electrode with organic electronic ion pumps (Fig. 1e)^41^. The size of the recording electrodes was 20 µm × 20 µm, and each of the electrodes was connected to an organic electronic ion pump with a 20 µm-wide outlet. Since the sizes of the recording electrodes and the outlets were on the same scale as single neuronal cells, an in vitro demonstration showed that the multifunctional probe allowed chemical modulation and electrical recording at a single-cell resolution. Nevertheless, the size of several hundred micrometers makes it challenging for in vivo neural recording and modulation with minimal insertion footprints and inflammatory responses. Another limitation is that the 32 outlets shared one reservoir, and it is difficult to individually control each sensing/delivery site.

## Multifunctional neural probes for neural recording and electrical stimulation

Electrical stimulation has been widely used in the restoration of sensory and motor functions^42^, as well as in the construction of brain–computer interfaces^43^. During electrical stimulation, electrical pulses are sent through stimulating electrodes to the targeted regions in the brain^44^. Electrical stimulation can induce charge redistribution at the electrode/neural interfaces through non-Faradic reactions or Faradaic reactions, leading to the depolarization or hyperpolarization of neurons.

**Fig. 2:**
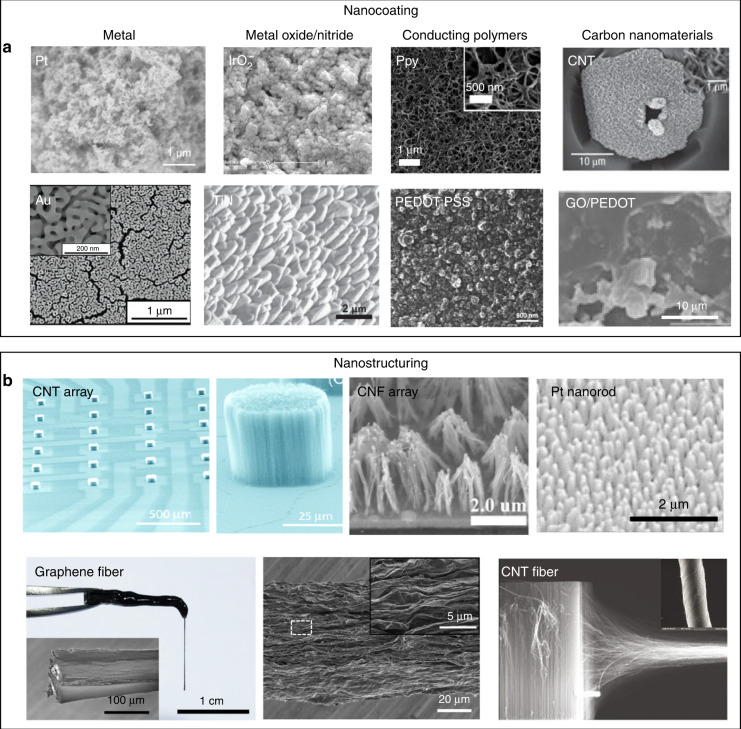
Examples of coated and structured stimulating microelectrodes with increased CIC. **a** Increasing CIC with surface coating of metal, metal oxide, conducting polymers, and carbon nanomaterials. Reproduced with permission from ref. ^45^. (Pt), copyright 2020, American Chemical Society; ref. ^46^. (Au), copyright 2017, John Wiley and Sons; ref. ^47^. (IrO_2_), copyright 2016, American Chemical Society; ref. ^48^. (TiN), copyright 2018, Elsevier; ref. ^51^. (Ppy). Copyright 2011, The Royal Society of Chemistry; ref. ^52^. (PEDOT). Copyright 2019, American Chemical Society; ref. ^53^. (CNT), copyright 2008, Nature Publishing Group; ref. ^50^. (PEDOT/GO), copyright 2019, Elsevier. **b** Increasing CIC with surface engineering to fabricate nanopillar arrays or microfibers with nanowrinkles. Reproduced with permission from ref. ^53^. (CNT array), copyright 2006, American Chemical Society; ref. ^55^. (Pt nanorods), copyright 2019, American Chemical Society; ref. ^56^. (Graphene microfiber), copyright 2020, Nature Publishing Group; ref. ^59^. (CNT yarn), copyright 2018, Nature Publishing Group

The stimulation efficiency of an electrode is determined by its charge injection capacity (CIC), which is the amount of charge that can be injected into brain tissue without inducing any irreversible chemical reactions at the surface of the stimulating electrode. To achieve increased CIC, attempts have been made to improve the specific surface areas of stimulating electrodes by coating them with porous nanomaterials, such as Pt^28,45^, gold^46^, IrO_2_^47^, TiN^48^, carbon nanotubes (CNTs)^49^, and graphene^50^ (Fig. 2a). In addition, pseudocapacitive conducting polymers, such as PPy^51^ and PEDOT^52^, have also been exploited as the coating layer to improve the CIC of stimulating electrodes (Fig. 2a). The introduction of these rough layers can increase the effective surface area of the stimulating electrodes, resulting in improved CIC. Another strategy to improve the CIC is through structural engineering of stimulating electrodes. For example, stimulating electrodes consisting of arrays of CNTs^53^, carbon nanofibers^54^ and porous Pt nanorods^55^ have been developed and exhibited improved stimulation efficiency compared to their planar counterparts (Fig. 2b). In addition, fiber electrodes, such as graphene fibers^56,57^ and CNT fibers^58,59^, have also been developed for electrical stimulation. The numerous nanowrinkles on these fibers could significantly increase their effective surface area, leading to an improved stimulation efficiency. Moreover, the small cross-sectional areas of the fiber-based stimulating electrodes also led to reduced traumatic implantation damage to the brain tissue^56^.

To investigate the response of neurons to electrical stimulation, multifunctional neural electrodes have been developed by integrating stimulating electrodes with recording electrodes. For example, Shi et al. developed an ultrathin multifunctional neural probe with integrated recording and stimulating electrodes and used a resorbable silk substrate as a temporary carrier^17^. After the dissolution of the silk substrate, the ultrathin multifunctional neural probe formed a biocompatible and conformal interface with the brain, enabling simultaneous neural activity modulation and recording with high spatiotemporal resolution. In addition, electrical modulation/recording interfaces with other novel functions have been developed, including transient electronics that can degrade after use^60^, self-healable electronics that can self-assemble on the static nerve^61^ and morphing electronics that can adapt their structure to a growing static nerve^62^.

Although neural activity can be effectively modulated by electrical stimulation, there are still several major challenges to be addressed: (1) Electrical stimulation leads to an electrical artifact in the recording channels that has a signal strength orders of magnitude higher than that of the recorded action potentials. (2) Electrical stimulation does not have cell type specificity. (3) During repeated stimulations, electrodes may undergo irreversible chemical reactions, resulting in decreased stimulation efficiency.

## Multifunctional neural probes for simultaneous neural recording and optical modulation

Optogenetics can enable millisecond-scale neural modulation in defined types of cells in behaving animals and has greatly advanced our understanding of neural circuit function^63^. In optogenetics, genes encoding light-activated ion channels/pumps, i.e., opsins, are expressed in specific types of cells^64^. Upon the absorption of light with the correct wavelength, these ion channels/pumps open and allow the inflow of Na^+^ or Cl^−^, leading to the depolarization (activation) or hyperpolarization (silencing) of neurons. This section discusses multifunctional neural interfaces for combined electrophysiological recording and optogenetic modulation.

### Multifunctional neural probes for electrocorticography (ECoG) recording/modulation

**Fig. 3:**
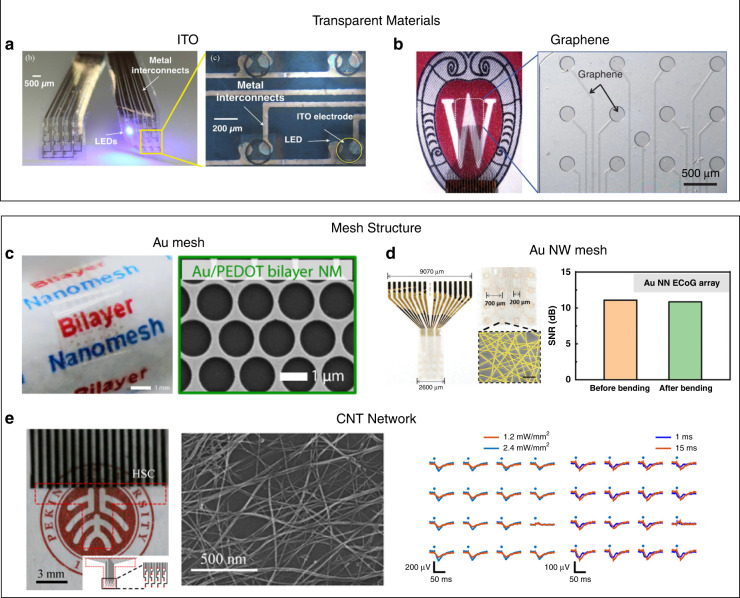
Examples of transparent ECoG electrodes for optogenetic stimulation. **a** A transparent ITO electrode array for optical stimulation and neural recording. Reproduced with permission from ref. ^67^. Copyright 2013, IEEE Xplore. **b** A transparent graphene electrode array for simultaneous optical stimulation and neural recording. Reproduced with permission from ref. ^68^. Copyright 2014, Nature Publishing Group. **c** Gold mesh electrode arrays fabricated by using sacrificial polystyrene spheres as templates. Reproduced with permission from ref. ^71^. Copyright 2018, American Association for the Advancement of Science. **d** Gold NW mesh electrodes and their stable recording performance under mechanical deformation. Reproduced with permission from ref. ^72^. Copyright 2020, John Wiley and Sons. **e** Transparent CNT spider-web electrode array for neural recording under optogenetic modulation. Reproduced with permission from ref. ^73^. Copyright 2018, American Chemical Society

ECoG electrodes can record from a large cortical surface^6^ and are less invasive than depth electrodes^65^. Therefore, combining ECoG electrodes with optogenetic techniques can provide large-scale mapping of physiological neural signals under optical stimulation. However, the opaque metallic conductive materials in conventional devices prevent direct optical stimulation at electrode-tissue interfaces^66^. To address this issue, indium tin oxide (ITO) has been used to fabricate transparent ECoG electrodes for optogenetic modulations because of its high transparency and conductivity. For example, Kwon et al. fabricated a transparent ITO electrode array on a parylene C substrate and integrated it with microLED-based light delivery units (Fig. 3a)^67^. The ITO electrode array could record light-evoked signals from the rat visual cortex transfected with AAV-hSyn-hChR2(H134R)-mCherry.

ITO-based electrodes usually suffer from rapidly decreased performance under mechanical deformation because of the brittleness of ITO. On the other hand, graphene has been considered to be a promising material for transparent ECoG electrodes because of its high transparency, excellent flexibility, and good biocompatibility^68–70^. Park et al. fabricated a transparent ECoG electrode array that was composed of four stacked graphene monolayers. The device was termed the carbon-layered electrode array (CLEAR) (Fig. 3b)^68^. The CLEAR device exhibited a light transmittance of approximately 90%, which enabled effective stimulation of opsin-expressing neurons underneath the graphene electrodes. Thunemann et al. further developed a graphene microelectrode array with no light-induced artifact by using residue- and contamination-free single-layer graphene as the electrode material^69^.

Graphene has limited conductivity compared to metals. Therefore, graphene electrodes with reduced sizes usually have high impedance, limiting their applications in high spatial resolution neural recording and manipulation. Recently, transparent metallic electrodes with a mesh- or grid-type structure design have been developed for simultaneous optogenetic stimulation and electrical recording. The large void areas in these mesh- and grid-type metallic electrodes allow efficient light transmission, leading to high transparence of the electrodes. Qiang et al. fabricated a transparent mesh electrode by patterning gold electrodes and connection lines with polystyrene spheres (Fig. 3c)^71^. The mesh-type gold electrodes showed no artifact under optical stimulation, enabling high-fidelity electrical recordings of light-evoked activities in an awake mouse. Seo et al. fabricated a transparent Au nanowire (NW) microelectrode array with a polymer fiber network as the shading mask (Fig. 3d)^72^. The microelectrode array had a high transparency of 87% and a low impedance of 18.6 Ω at 1 kHz, which enabled 2D mapping of light-evoked signals in transgenic mice. However, metal-based microelectrode arrays usually suffer from low stretchability, which limits their applications in mechanically active environments. To address this issue, Zhang et al. developed a transparent and stretchable ECoG electrode array with a CNT spider-web network as the electrode material and PDMS as the substrate (Fig. 3e)^73^. The ECoG electrode array exhibited a high stretchability, and the impedance increased by only 26% at a tensile strain of 20%. As a result, the device showed no performance change even after being hit by a falling rod.

### Multifunctional neural probes for depth recording/modulation

Compared to ECoG electrodes, depth electrodes can allow neural activity recordings at single spike resolution. This section discusses depth electrode-based recording/optical modulation interfaces with various light delivery units, including optical fiber, LEDs, upconverting nanoparticles (UCNPs), and thermally drawn fibers.

#### Neural modulation with optical fiber and LEDs

**Fig. 4:**
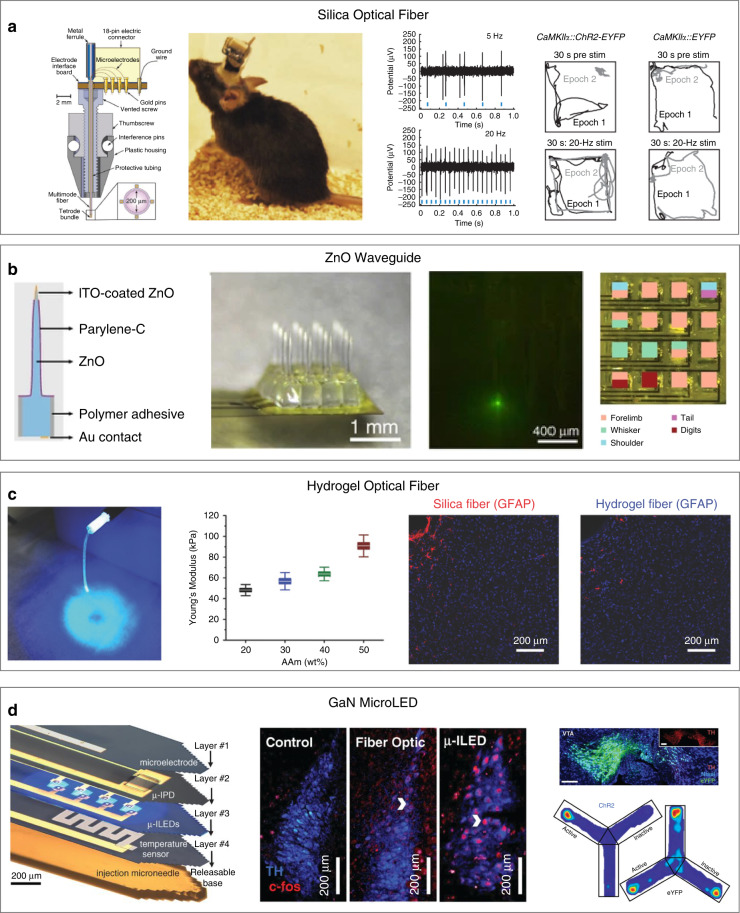
Examples of optical modulation/recording interfaces with optical fibers and microLEDs. **a** A microwire electrode bundle surrounded by four optical fibers for optical neural modulation of freely behaving mice. Reproduced with permission from ref. ^19^. Copyright 2012, Nature Publishing Group. **b** A transparent ZnO microprobe array for optogenetic neural manipulation. Reproduced with permission from ref. ^77^. Copyright 2015, Nature Publishing Group. **c** A soft alginate-PAAm hydrogel optical fiber with low Young’s modulus and reduced inflammatory response of the brain tissue. Reproduced with permission from ref. ^79^. Copyright 2018, John Wiley and Sons. **d** A multilayer, injectable optical modulation/recording interface based on µ-ILEDs for optical neural modulation. Reproduced with permission from ref. ^80^. Copyright 2013, AAAS

Optical fibers have been widely used as the light source for neural modulation in both in vitro and in vivo studies^74–76^. In 2012, Anikeeva et al. developed an optrode device by manually attaching four tetrode bundles of nickel and chromium alloy wire onto an optical fiber (Fig. 4a)^18^. The device had a light weight of only 2 g and a height of 22 mm and could record light-evoked responses of medial prefrontal cortex neurons in freely moving mice.

Optical modulation/recording interfaces with assembled optical fibers and recording microelectrodes typically involve manual assembly of two or more separate material blocks, resulting in a large physical size. In addition, these assemblies have limited spatial resolution. To address these issues, Lee et al. developed a 4 × 4 micro-optoelectrode array (MOA) with transparent ZnO micropillars as both waveguides and conductive recording electrodes (Fig. 4b)^77^. Owing to the micrometer-sized output apertures, the MOA devices detected spiking responses at light power as low as 1.76 ± 1.79 μW. Moreover, the MOA enabled efficient neural modulation at high spatial resolution. In another example, Zou et al. developed a multifunctional neural probe by immersing an array of ultraflexible microelectrode filaments and an optical fiber into a viral vector containing a polyethylene glycol (PEG) bath and withdrawing it into ambient air^78^. During the withdrawing process, the PEG quickly solidified, resulting in an implantable AAV delivery optrode. After implantation into the brain, the virus vectors were released after PEG dissolution, resulting in highly localized transduction of neurons near the microelectrodes. The AAV-delivery optrode allowed simultaneous neural activity recording and modulation of spatially defined neuronal populations for 3 months.

Conventional optical fibers are mainly based on silica, whose Young’s modulus is approximately six orders of magnitude higher than that of the brain tissue. This large mechanical mismatch causes significant implantation damage to the brain tissue, which subsequently induces neuronal loss at the implantation sites. To address this issue, Wang et al. developed a soft alginate-polyacrylamide hydrogel optical fiber for neural modulation (Fig. 4c)^79^. The Young’s modulus of the hydrogel optical fiber was only tens of kPa, similar to that of brain tissues (≈1 kPa). As a result, the soft device elicited a greatly reduced chronic inflammatory response compared with its rigid counterpart. However, swollen hydrogel optical fibers usually show lower light delivery efficiency at increased depth than conventional optical fibers due to increased propagation loss. Therefore, it is difficult for hydrogel optical fibers to be used in deep brain stimulation.

Although optical fiber-based light stimulation allows neural activity modulation with high temporal resolution, the large sizes of the optical fibers can cause greater damage upon insertion into the brain tissue. In addition, the tethered nature of the optical fibers can restrict animals’ natural behavior. Compared to optical fibers, microLEDs are smaller, less expensive, and available at various wavelengths. In particular, the small sizes of microLEDs can allow their precise integration onto neural probes^80^. In addition, microLEDs can be wirelessly powered and allow tetherless light delivery^81^. Therefore, multifunctional neural interfaces with integrated microLEDs can allow precise neural modulation in awake, behaving rodents through optogenetic manipulation. Kim et al. developed a wireless, flexible, and layered multifunctional interface that consists of four microscale GaN LEDs (µ-ILEDs), a Pt microelectrode, a photodetector, and a temperature sensor (Fig. 4d)^80^. The cross-section of µ-ILEDs was only 50 μm × 50 μm. Therefore, light could be delivered to the targeted brain region at cellular-scale precision. In addition, the small physical size and mechanical flexibility of the multifunctional probe resulted in greatly reduced neuronal loss compared to a metal cannula and fiber optics.

#### Neural modulation with UCNPs

**Fig. 5:**
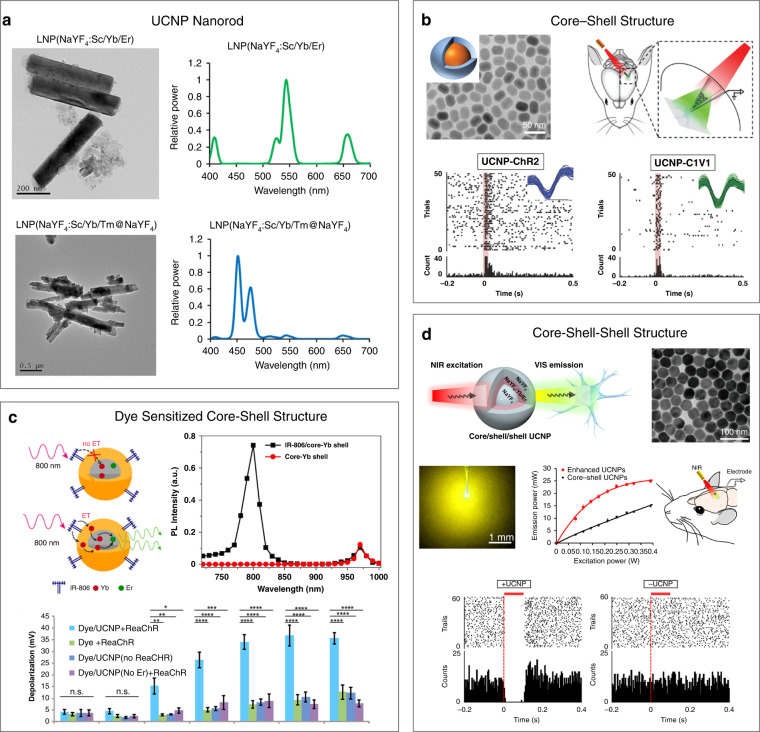
Optogenetic neural activity modulation with UCNPs. **a** Lanthanide-doped nanorods with different upconversion emission spectra. Reproduced with permission from ref. ^83^. Copyright 2015, Nature Publishing Group. **b** Core–shell UCNPs for neural activity modulation in living animals. Reproduced with permission from ref. ^86^. Copyright 2017, John Wiley and Sons. **c** Dye-sensitized core-shell UCNPs with enhanced upconversion emission efficiency for neural activity manipulation. Reproduced with permission from ref. ^84^. Copyright 2016, American Chemical Society. **d** Core-shell-shell UCNPs with enhanced upconversion emission efficiency for neural activity inhibition. Reproduced with permission from ref. ^88^. Copyright 2018, American Chemical Society

Most photosensitive opsins can only be activated with visible light. However, visible light has limited transmission depth in the brain. As a result, light-delivery devices, such as optical fibers or microLEDs, need to be implanted into deep brain tissues. By contrast, near-infrared (NIR) (650–1450 nm) light can penetrate deep brain tissues. Therefore, the development of systems that can convert highly tissue-penetrative NIR light to visible light represents a promising strategy for low-invasive optical neural stimulation. UCNPs are composed of rare-earth components and can absorb low-energy NIR light and emit high-energy visible light through a multiphoton absorption process^82^. Therefore, UCNPs are promising candidates for low-invasive neural modulation. In particular, lanthanide-doped UCNPs with emission spectra from blue to yellow have been synthesized and used to manipulate the neural activities of cells^83,84^, worms^85^, and living rodents^86–90^. For example, Hososhima et al. synthesized NaYF_4_:Sc/Yb/Er and NaYF_4_:Sc/Yb/Tm@NaYF_4_ nanorods, which could absorb NIR light and emit green and blue light, respectively (Fig. 5a)^83^. The emitted visible light-induced the photoreactive response of the nearby ND7/23 cells expressing CHR opsins. Chen et al. injected UCNPs into ventral tegmental area (VTA) neurons that expressed light-sensitive proteins^89^. Tissue-penetrating NIR light could be locally converted by UCNPs to visible light to evoke dopamine release in VTA DA neurons.

UCNPs usually have a low light emission efficiency due to surface quenching. To address this issue, Lin et al. synthesized core-shell UCNPs with Tm^3+^- or Er^3+^-doped NaYF_4_ as the core and NaYF_4_ as the shell and applied the UCNPs to NIR-based optogenetic modulation in rodents (Fig. 5b)^86^. The core-shell structure design could effectively block surface quenchers, leading to improved emission efficiency. In another example, they further developed a robotic laser projection system for tetherless optogenetic modulation in different rat brain regions and complex animal behavior control^87^. Wu et al. further synthesized a type of dye-sensitized core-shell UCNP with NaYF_4_:Yb^3+^:Er^3+^ as the core, NaYF_4_:Yb^3+^ as the shell and IR-806 as the dye sensitizer (Fig. 5c)^84^. The dye-sensitized core-shell UCNPs showed ∼1000 fold upconversion luminescence enhancement compared to the same UCNPs without dye sensitizing. As a result, the dye-sensitized core-shell UCNPs enabled robust activation of ReaChR-expressing hippocampal neurons. Lin et al. synthesized NaYF_4_-based UCNPs with a unique core-shell-shell structure^88^. The UCNPs consisted of a β-NaYF_4_ core, a β-NaYF_4_:Yb/Er shell layer, and an outer β-NaYF_4_ shell layer (Fig. 5d). The outer β-NaYF_4_ provided spatial confinement to the Yb lattice, which enabled the elimination of Yb-associated concentration quenching. Compared to core-shell UCNPs, the core-shell-shell UCNPs exhibited an almost threefold enhanced emission intensity at ~540–570 nm, enabling reliable wireless modulation of eNpHR-expressing neurons in rats.

#### Fiber-based multifunctional neural probes

**Fig. 6:**
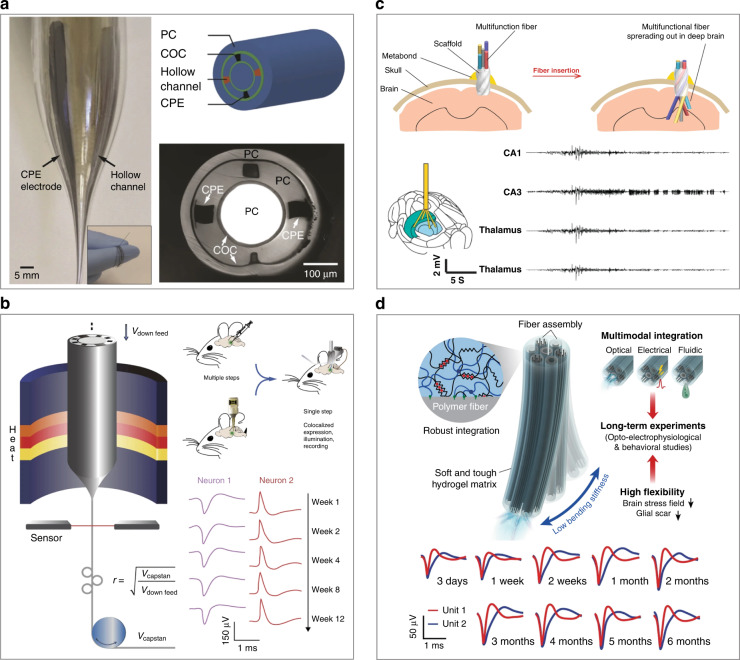
Examples of fiber-based multifunctional neural interfaces. **a** A thermally drawn fiber-based multifunctional neural interface and its optical image. Reproduced with permission from ref. ^91^. Copyright 2015, Nature Publishing Group. **b** Fiber-based multifunctional neural interfaces for one-step optogenetics and chronic neural activity recording. Reproduced with permission from ref. ^92^. Copyright 2017, Nature Publishing Group. **c** A spatially expandable fiber-based multifunctional neural interface for simultaneous neural activity recording and modulation across distant brain regions. Reproduced with permission from ref. ^93^. Copyright 2020, Nature Publishing Group. **d** A hydrogel matrix-held fiber-based multifunctional neural interface for chronic neural activity recording and modulation. Reproduced with permission from ref. ^94^. Copyright 2021, Nature Publishing Group

Multifunctional fiber probes have been demonstrated for low-invasive neural activity recording and modulation. For example, Canales et al. developed an all-polymer fiber probe by a thermal drawing process for combined optical stimulation, drug delivery and neural recording^91^. The multifunctional fiber consisted of a polycarbonate (PC) waveguide, a cyclic olefin copolymer light confinement layer, an additional PC encapsulation layer, and conductive polyethylene recording electrodes (Fig. 6a). The PC waveguide was surrounded with conductive polyethylene recording electrodes and microfluidic channels for drug delivery. Owing to its small cross-section, the multifunctional fiber probe elicited minimal inflammatory responses of the brain tissue and allowed optogenetic stimulation/electrical recording in freely moving transgenic mice for over 2 months.

Park et al. further introduced graphite into conductive polyethylene to reduce the resistance of the recording electrodes in thermally drawn multifunctional fibers^92^. As a result, the size of the recording electrodes was reduced to 20–25 µm in diameter. Moreover, the microchannels in the multifunctional fiber allowed the delivery of virus vectors into the mouse brain. Thus, the multifunctional probes could allow virus injection and optrode implantation through a single-step surgery (Fig. 6b). Recently, Jiang et al. developed a spatially expandable multifunctional fiber electrode by inserting an array of multifunctional fibers into the hollow channels of a helical scaffolding fiber^93^. After implantation, the multifunctional fiber electrode extruded out of the scaffold and expanded to reach the multiple targeted regions through a single implantation (Fig. 6c). As a result, the device allowed simultaneous recording and optical/chemical neural modulation across distant regions. Park et al. further fabricated a compliant multifunctional probe by integrating fiber microelectrodes within a hydrogel matrix (Fig. 6d)^94^. The bending stiffness of the hybrid probe was only 0.42 N m^−1^. Therefore, the hybrid probe induced minimal foreign body responses and enabled stable tracking of single neurons in freely moving mice for over 6 months after implantation.

## Conclusion and prospects

Multifunctional neural probes have greatly advanced our understanding of brain functions. This review summarizes the progress of multifunctional neural probes with different neural modulation modalities, including chemical stimulation, electrical stimulation, and optogenetic stimulation. Although substantial progress has been made in multifunctional neural probes, there are still significant challenges to be addressed, including the following: (i) Modern multifunctional neural probes are mainly composed of rigid stimulation units with relatively large footprints (e.g., microfluidics and optical fibers). The rigid nature and large footprints of the stimulation units can induce elevated tissue damage and inflammatory responses in the brain, limiting the use of these multifunctional probes in chronic studies. Therefore, it is highly desirable to develop mechanically compliant and miniature multifunctional neural probes for long-term stable neural recording and modulation. (ii) It is still challenging for modern neural modulation techniques to achieve type-specific modulation at single-neuron resolution. For instance, optogenetic modulation and chemical modulation can achieve cell-specific modulation but are limited in spatial resolution due to light propagation or chemical diffusion. Therefore, the development of new neural modulation techniques with combined high spatiotemporal resolution and cell specificity is highly desirable, especially for the study of neural circuit mechanisms. (iii) Neural modulation, including electrical stimulation and light stimulation, can result in changes in the microenvironment surrounding neurons, including temperature and pH. It is thus desirable to integrate multifunctional neural probes with other functionalities, such as temperature monitors and pH sensors. Addressing these challenges relies on not only the materials and structure optimization of neural interfaces but also multidisciplinary scientific collaborations from scientists and engineers of materials science, electronics, mechanical engineering, and neuroscience.

## Supplementary information

Table of Contents
