# Supplementary material for: Recent developments in multifunctional neural probes for simultaneous neural recording and modulation
Source: Microsyst Nanoeng. 2023 Jan 4;9:4. doi: 10.1038/s41378-022-00444-5 (PMC9810608; doi:10.1038/s41378-022-00444-5)

**Recent development in multifunctional neural probes for simultaneous neural recording and modulation**

Hongbian Li^1^, Jinfen Wang^1^, Ying Fang^1,2*^

^1^CAS Key Laboratory for Biomedical Effects of Nanomaterials and Nanosafety, CAS Center for Excellence in Nanoscience, National Center for Nanoscience and Technology, Beijing, 100190, China

^2^CAS Center for Excellence in Brain Science and Intelligence Technology, Institute of Neuroscience, Chinese Academy of Sciences, Shanghai 200031, China

Table of Contents


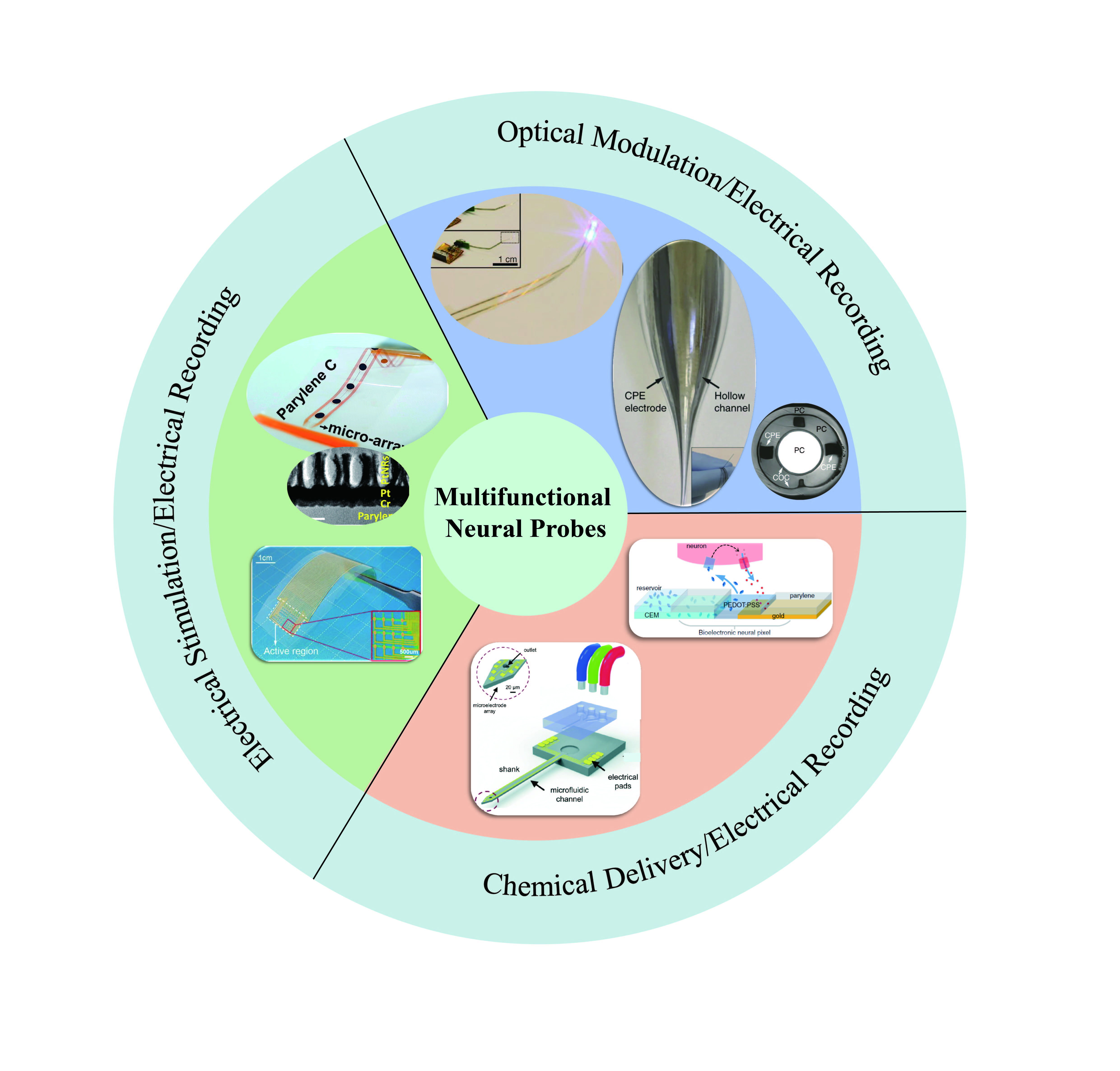

Supplement: Supplementary file 1 — Table of Contents [file 41378_2022_444_MOESM1_ESM.docx]
